# The hepatokine TSK maintains myofiber integrity and exercise endurance and contributes to muscle regeneration

**DOI:** 10.1172/jci.insight.154746

**Published:** 2022-02-22

**Authors:** Qiuyu Wang, Xiaoxue Qiu, Tongyu Liu, Cheehoon Ahn, Jeffrey F. Horowitz, Jiandie D. Lin

**Affiliations:** 1 Life Sciences Institute and Department of Cell & Developmental Biology, Michigan Medicine, and; 2 Substrate Metabolism Laboratory, School of Kinesiology, University of Michigan, Ann Arbor, Michigan, USA.

**Keywords:** Muscle Biology, Growth factors

## Abstract

Mammalian skeletal muscle contains heterogenous myofibers with different contractile and metabolic properties that sustain muscle mass and endurance capacity. The transcriptional regulators that govern these myofiber gene programs have been elucidated. However, the hormonal cues that direct the specification of myofiber types and muscle endurance remain largely unknown. Here, we uncover the secreted factor Tsukushi (TSK) as an extracellular signal that is required for maintaining muscle mass, strength, and endurance capacity and that contributes to muscle regeneration. Mice lacking TSK exhibited reduced grip strength and impaired exercise capacity. Muscle transcriptomic analysis revealed that TSK deficiency results in a remarkably selective impairment in the expression of myofibrillar genes, characteristic of slow-twitch muscle fibers, that is associated with abnormal neuromuscular junction formation. AAV-mediated overexpression of TSK failed to rescue these myofiber defects in adult mice, suggesting that the effects of TSK on myofibers are likely restricted to certain developmental stages. Finally, mice lacking TSK exhibited diminished muscle regeneration following cardiotoxin-induced muscle injury. These findings support a crucial role of TSK as a hormonal cue in the regulation of contractile gene expression, endurance capacity, and muscle regeneration.

## Introduction

Skeletal myofibers in mammals exhibit remarkable heterogeneity and plasticity (1–3). Based on the expression of myosin heavy chain (MYH) isoforms and their metabolic properties, skeletal muscle fibers can be classified as slow-twitch, type I, and as fast-twitch, type II (IIa, IIb, and IIx), fibers. Type I and IIa myofibers have relatively high mitochondrial content and oxidative capacity, whereas type IIb and IIx myofibers rely primarily on glycolytic metabolism for energy production. The slow-twitch myofibers are typically more fatigue resistant and play a crucial role in sustaining the endurance capacity of skeletal muscle. Previous work has established several signaling pathways, including calcineurin and AMPK (2, 4, 5), and transcriptional regulators in the control of the oxidative and glycolytic muscle fiber gene programs, including the PGC-1 family of transcriptional coactivators (PGC-1α, PGC-1β) (6–8), the chromatin regulator BAF60c (9), nuclear hormone receptors (ERRα, ERRγ, PPARδ) (10), and the MEF2 transcription factors (2). Motor nerve activity and signaling via the neuromuscular junction are important drivers of myofiber determination, metabolism, and function. However, the extracellular signals and hormonal cues that specify the metabolic and contractile properties of skeletal myofibers remain poorly defined.

Tsukushi (TSK) is a leucine-rich repeat–containing protein that was initially identified as a regulator of brain development (11–13). TSK physically interacts with and modulates the activity of several growth factors, including the TGF-β, FGF, and Wnt families of extracellular ligands (14–16). We and others recently demonstrated that TSK is abundantly expressed by the liver, which provides a major source of circulating TSK (17, 18). Hepatic TSK expression and its plasma levels are highly responsive to nutritional status and environmental cues (18–20). Interestingly, altered TSK levels have been observed in several disease conditions in humans, including metabolic disorders, hyperthyroidism, and lung cancer (21–23). Mice lacking TSK are resistant to diet-induced obesity, insulin resistance, and hepatic steatosis, in part through activation of brown fat sympathetic innervation and thermogenesis (18, 20). Beyond its effects on adipose tissue, recent work demonstrates that TSK regulates feeding and energy expenditure via its crosstalk with central melanocortin signaling (19). These findings implicate TSK as a versatile regulator of tissue development and metabolic homeostasis. In this study, we demonstrate that TSK serves an indispensable role in the regulation of several aspects of skeletal muscle biology, including slow-twitch myofiber development, muscle mass and strength, exercise endurance, and muscle regeneration.

## Results

### TSK is required for maintaining skeletal muscle mass and endurance capacity.

**Figure 1:**
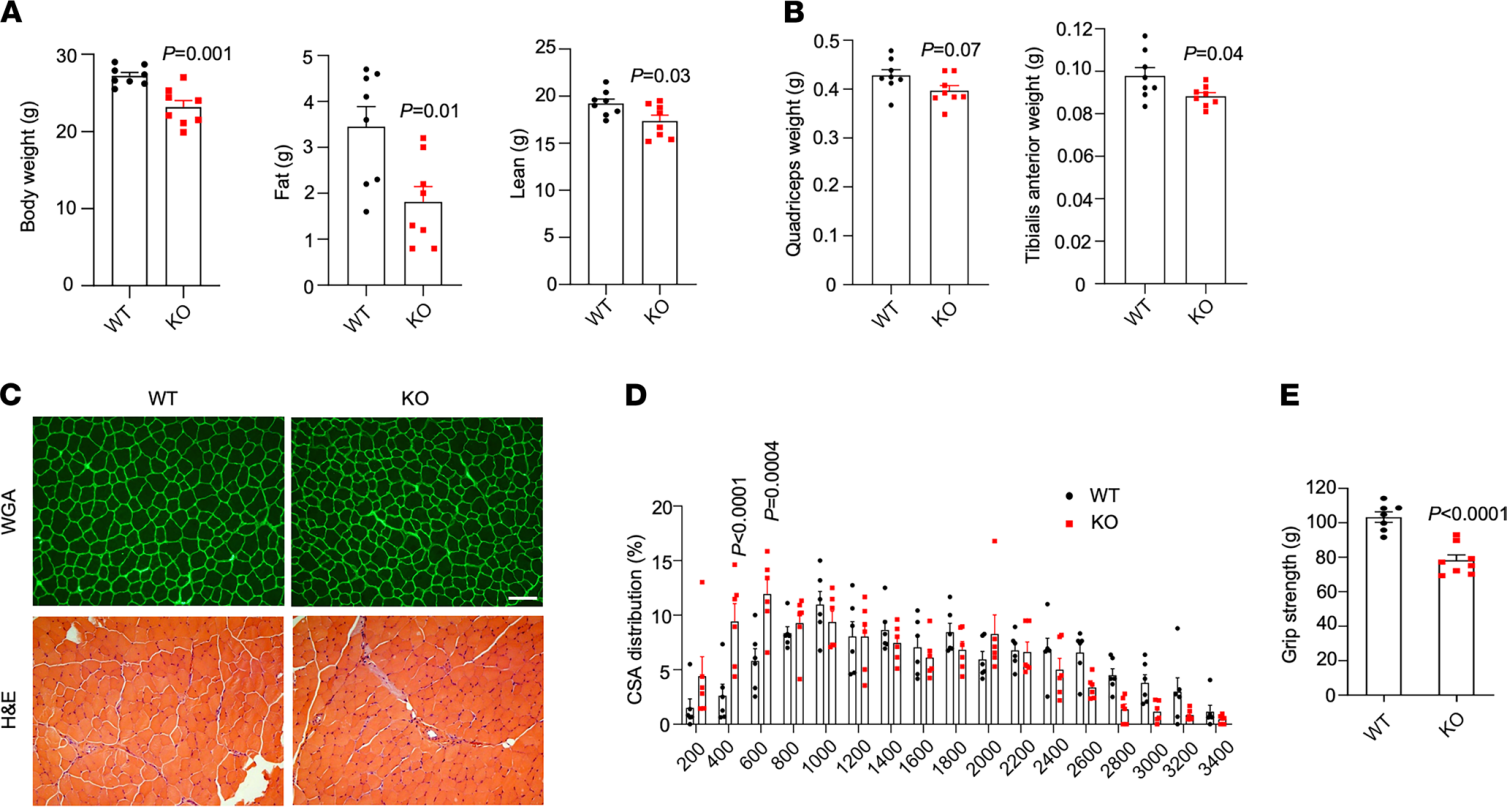
Regulation of skeletal muscle mass and fiber size by TSK. (**A**) Body weight, fat mass, and lean mass in WT (*n* = 8) and TSK KO (*n* = 8) mice. (**B**) Quadriceps and TA muscle weight. (**C**) Immunofluorescence (top, WGA) and H&E (bottom) staining of TA muscle sections. Scale bar: 100 μm. (**D**) Quantitation of muscle fiber size distribution. (**E**) Forelimb grip strength in WT (*n* = 7) and TSK KO (*n* = 8) mice. Data represent mean ± SEM and were analyzed by 2-tailed unpaired Student’s *t* test (**A**, **B**, and **E**) and 2-way ANOVA with post hoc analysis using Tukey’s test (**D**).

In previous studies, we demonstrated that mice deficient in TSK exhibited remarkable resistance to high-fat diet–induced (HFD-induced) obesity and its associated metabolic disorders, including insulin resistance, adipose dysfunction, and hepatic steatosis (18–20). TSK-KO mice were slightly smaller than WT littermates when fed chow diet and gained significantly less weight following HFD feeding (Supplemental Figure 1, A and B; supplemental material available online with this article; https://doi.org/10.1172/jci.insight.154746DS1). Body composition analysis in mice fed HFD for 2 weeks revealed that both fat mass and lean mass were significantly decreased by TSK deficiency (Figure 1A). Accordingly, quadriceps and tibialis anterior (TA) muscle mass was lower in TSK-null mice than WT littermate control (Figure 1B). To determine whether TSK deficiency alters myofiber sizes, we performed H&E histological staining and WGA lectin immunofluorescence staining on TA muscle sections from WT and TSK-null mice. While the overall appearance of myofibers was similar between 2 groups, there was a notable shift toward smaller muscle fibers in mice lacking TSK (Figure 1, C and D). Measurements of grip strength indicated that, compared with control, TSK-null mice had significantly reduced forelimb force generation (Figure 1E and Supplemental Figure 1C). These findings raise the possibility that TSK signaling may play a previously unappreciated role in maintaining skeletal muscle mass and in other aspects of muscle biology.

**Figure 2:**
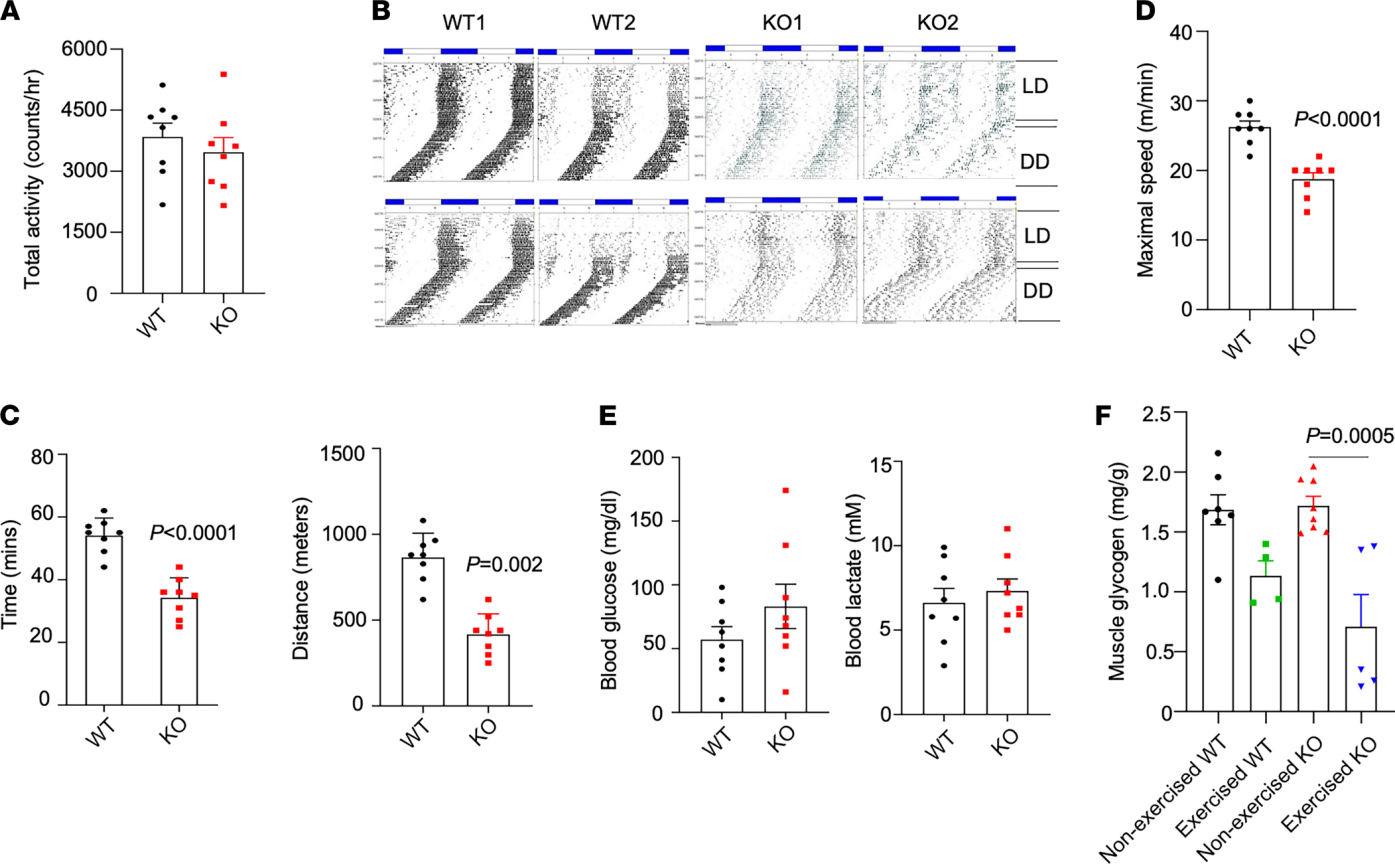
TSK inactivation impairs exercise endurance capacity in mice. (**A**) Total locomotor activity monitored by beam breaking using metabolic cages. (**B**) Voluntary wheel running during light-dark cycles (LD) and constant darkness (DD). Wheel running activity is indicated by blue bars in the graph. Traces of 2 representative WT and TSK-KO mice are shown. (**C**) Treadmill running time to exhaustion and running distance in WT (*n* = 8) and TSK KO (*n* = 8) mice. (**D**) Maximal speed during running. (**E**) Blood glucose and lactate levels following treadmill running. (**F**) Glycogen content in quadriceps muscles from nonexercised WT (*n* = 7) and TSK KO (*n* = 8) mice and a separate group (WT, *n* = 4; KO, *n* = 5) following treadmill running. Data represent mean ± SEM and were analyzed by a 2-tailed unpaired Student’s *t* test (**A** and **C**–**E**) or 1-way ANOVA with post hoc analysis using Tukey’s test (**F**).

We previously performed metabolic cage studies to assess the effects of TSK deficiency on food intake, energy expenditure, and locomotor activity (18). As shown in Figure 2A, total cage activity levels during light and dark cycles were comparable between control and TSK-null mice, indicating that TSK deficiency does not alter basal daily locomotor function in mice. To determine whether TSK inactivation affects muscle function under exertion, we performed voluntary wheel running and treadmill running studies. We individually housed control and TSK-null mice in cages equipped with a running wheel and recorded their running activities. As expected, most of the wheeling running activity was confined to the dark phase in both groups (Figure 2B). Following the switch from light/dark (LD) cycles to constant darkness (DD), there was a shift of activity patterns to earlier time, indicative of the slightly shorter than 24-hour free-running circadian clock period. This shift appeared comparable between 2 genotypes. In contrast, TSK-deficient mice exhibited markedly reduced activity levels, most noticeably during the dark phase, suggesting that TSK-KO mice may have impaired exercise capacity.

Because wheel running is voluntary in nature, the reduction of activity levels in TSK-null mice may result from factors other than muscle performance. We next performed treadmill running studies to more directly assess whether TSK deficiency alters exercise endurance capacity in mice. We subjected WT and TSK-KO mice to 3 training sessions on a rodent treadmill before our studies. Under these experimental conditions, control mice were able to perform treadmill running for an average of approximately 54 minutes and 866 meters in distance before reaching exhaustion (Figure 2C). Time to exhaustion and running distance were reduced by 37% and 52%, respectively, in the TSK-null cohort. TSK-KO mice achieved significantly lower maximal running speed under this protocol (Figure 2D). Plasma glucose and lactate concentration remained similar between 2 groups (Figure 2E). Since glycogen provides a major source of metabolic fuel for muscle contraction, we measured muscle glycogen content in nonexercised mice and a separate cohort following treadmill running. As shown in Figure 2F, both basal and postexercise glycogen content was comparable between control and TSK-KO mice. Together, these results demonstrate that TSK is required for sustaining exercise endurance in mice.

### TSK deficiency impairs slow-twitch myofiber development and neuromuscular junction formation.

**Figure 3:**
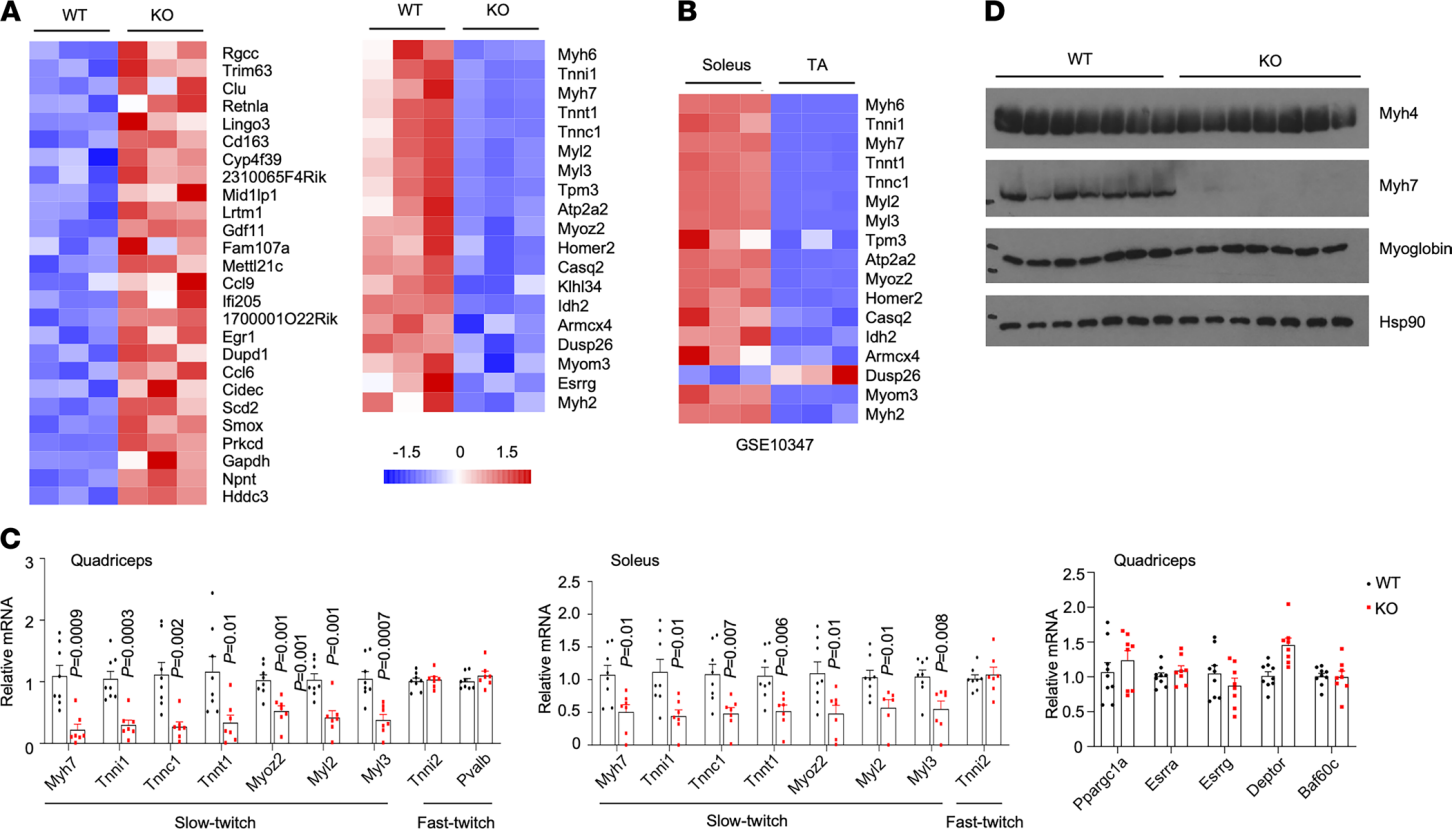
Regulation of skeletal muscle transcriptome by TSK. (**A**) RNA-Seq analysis of total quadriceps RNA from 3 pairs of WT and TSK-KO mice. Heatmaps of differentially expressed genes with over 1.5-fold upregulation (left) or downregulation (right) in TSK-KO muscle. (**B**) Heatmap illustrating mRNA expression of TSK-regulated muscle genes in soleus and TA based on the microarray data set GSE10347. (**C**) qPCR analysis of gene expression in quadriceps and soleus from WT (*n* = 8) and TSK-KO (*n* = 7) mice. (**D**) Immunoblots of total muscle lysates. Data in **C** represent mean ± SEM; 2-tailed unpaired Student’s *t* test.

To explore the mechanisms through which TSK regulates skeletal muscle function, we performed RNA-Seq on quadriceps from WT and TSK-KO mice. TSK deficiency does not appear to markedly alter the skeletal muscle transcriptome. We identified a total of 45 differentially regulated genes that exhibited over 1.5-fold changes in mRNA expression (Figure 3A). Among 26 genes upregulated in TSK-null muscle are those involved in chemokine signaling (*Ccl6*, *Ccl9*), inflammatory response (*Cd163*, *Ifi205*, *Retnla*), and cellular metabolism (*Cidec*, *Scd2*, *Gapdh*, *Smox*). Strikingly, many of the 19 downregulated genes correspond to genes encoding slow-twitch myofibrillar proteins, including MHC (*Myh2*, *Myh6*, *Myh7*), myosin light chain (*Myl2*, *Myl3*), slow-twitch troponin (*Tnnt1*, *Tnnc1*, *Tpm3*), and calcium regulation (*Atp2a2*, *Homer2*, *Casq2*). Most of these genes exhibited enriched expression in soleus, a muscle containing abundant slow-twitch myofibers, compared with TA muscle, which contains mixed muscle fibers (Figure 3B). Quantitative PCR (qPCR) analysis of gene expression confirmed decreased expression of these genes in quadriceps and soleus muscles from TSK-KO mice (Figure 3C). In contrast, fast-twitch fiber-enriched genes such as Tnni2 and Parvalbumin remained largely unaffected by TSK inactivation. Accordingly, protein levels of MYH7, a MHC-β enriched in the slow-twitch muscle fiber, were greatly reduced in quadriceps from mice lacking TSK (Figure 3D). Surprisingly, mRNA expression of known transcriptional regulators of myofiber determination and metabolism (*Ppargc1a*, *Esrra*, *Esrrg*, *Deptor*, *Baf60c*) remained largely unaltered. As such, these transcriptomic analyses revealed a remarkably selective role of TSK in maintaining slow-twitch myofiber gene expression.

**Figure 4:**
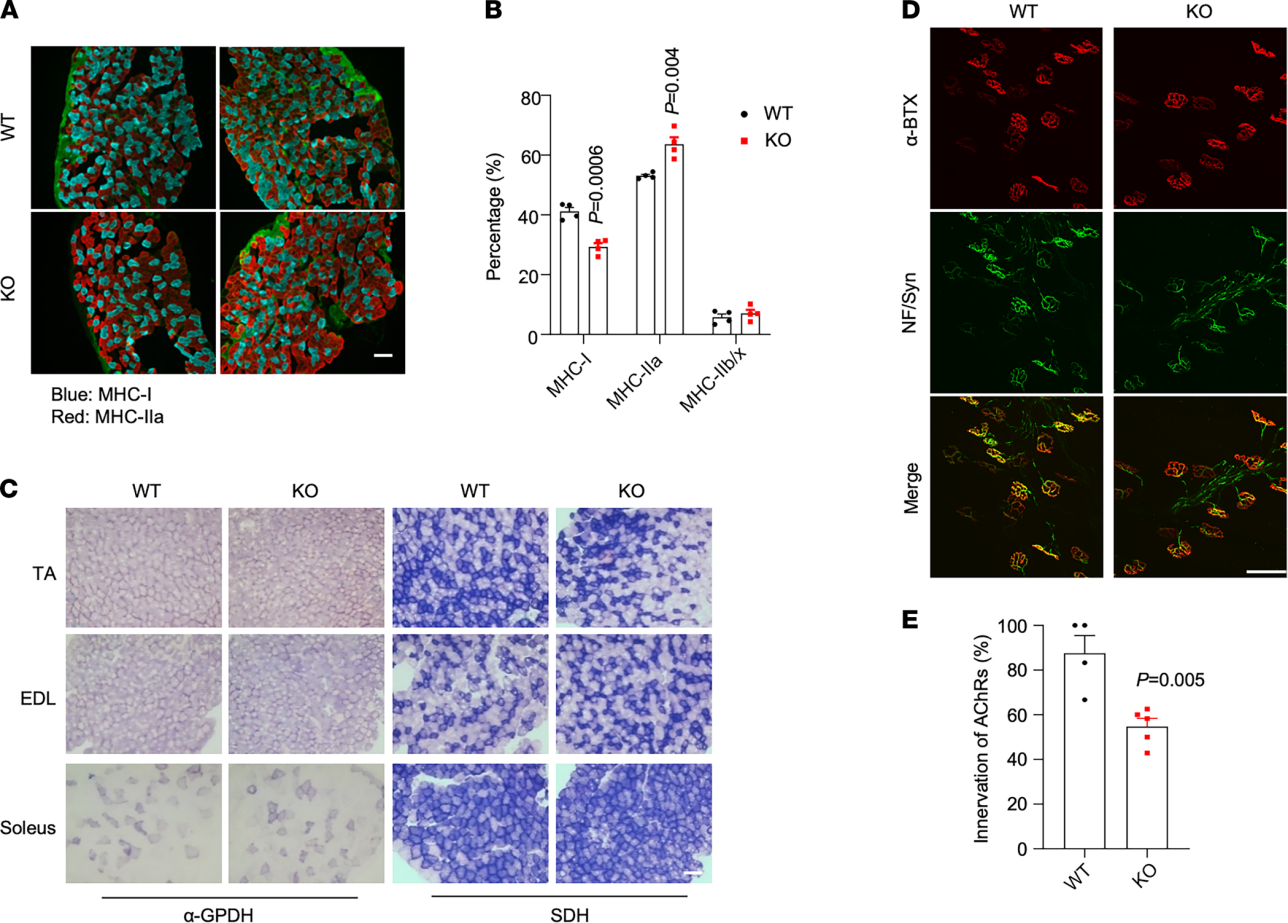
Regulation of muscle fiber type and neuromuscular junction by TSK. (**A**) Immunofluorescence staining for MHC-I and MHC-IIa in soleus. Scale bar: 100 μm. (**B**) Quantitation of type I, IIa, and IIb/x muscle fiber content in WT and TSK-KO soleus. (**C**) Histochemical enzymatic staining for α-GPDH and SDH. Scale bar: 100 μm. (**D**) Confocal images of immunofluorescence staining for α-BTX and anti-neurofilament/anti-synapsin (NF/Syn) on TA muscle sections. Scale bar: 100 μm. (**E**) Quantitation of neuromuscular innervation. Data in **B** and **E** represent mean ± SEM; 2-tailed unpaired Student’s *t* test.

Skeletal myofibers are heterogenous in their contractile function and metabolic properties (1–3). Slow-twitch type I myofibers typically contain abundant mitochondria, generate energy mainly from oxidative metabolism, and are more resistant to contraction-induced fatigue. Fast-twitch type IIa myofibers also have high oxidative capacity, whereas type IIb/x fibers are generally more reliant on glycolysis for energy production and fatigue-prone following muscle contraction. To further evaluate how TSK deficiency affects myofiber types and their metabolic function, we performed muscle fiber type analysis and histochemical enzymatic staining. As shown in Figure 4, A and B, TSK deficiency resulted in a significant decrease in the percentage of MHC-I^+^ muscle fibers in soleus with a corresponding increase in type IIa, but not IIb/x myofibers. These results are consistent with reduced mRNA expression of gene-encoding slow-twitch contractile proteins. Interestingly, histochemical enzymatic staining indicates that muscles from control and TSK-KO mice exhibited comparable enzymatic activity for α-glyceraldehyde 3-phosphate dehydrogenase (α-GPDH) and succinate dehydrogenase (SDH), which reflect glycolytic and oxidative energy metabolism, respectively (Figure 4C). We conclude that TSK deficiency selectively impairs the contractile aspect of myofiber phenotype of slow-twitch myofibers without grossly altering their glycolytic and oxidative capacity.

TSK has been demonstrated to regulate multiple neurodevelopment processes and adipose tissue sympathetic innervation (11, 12, 18). Given that myofiber gene expression is critically dependent on motor nerve activities, we next examined whether TSK deficiency may impair the integrity of neuromuscular junction and motor nerve coupling. Fluorescence-labeled α-botulinum toxin A (α-BTX) has been commonly used to label neuromuscular junctions. We observed similar staining patterns for α-BTX in control and TSK-KO muscle, suggesting that the formation of neuromuscular junctions per se remain largely unaffected by TSK inactivation (Figure 4D). In contrast, costaining with nerve fiber markers revealed that colocalization of nerve termini with neuromuscular junctions was diminished in TSK-KO mice compared with control (Figure 4E). Together, these observations illustrate a unique role of TSK in the regulation of contractile gene expression in slow-twitch myofibers and the maintenance of the integrity of muscle-nerve coupling.

### Skeletal muscle defects in mice lacking TSK are of a developmental origin.

**Figure 5:**
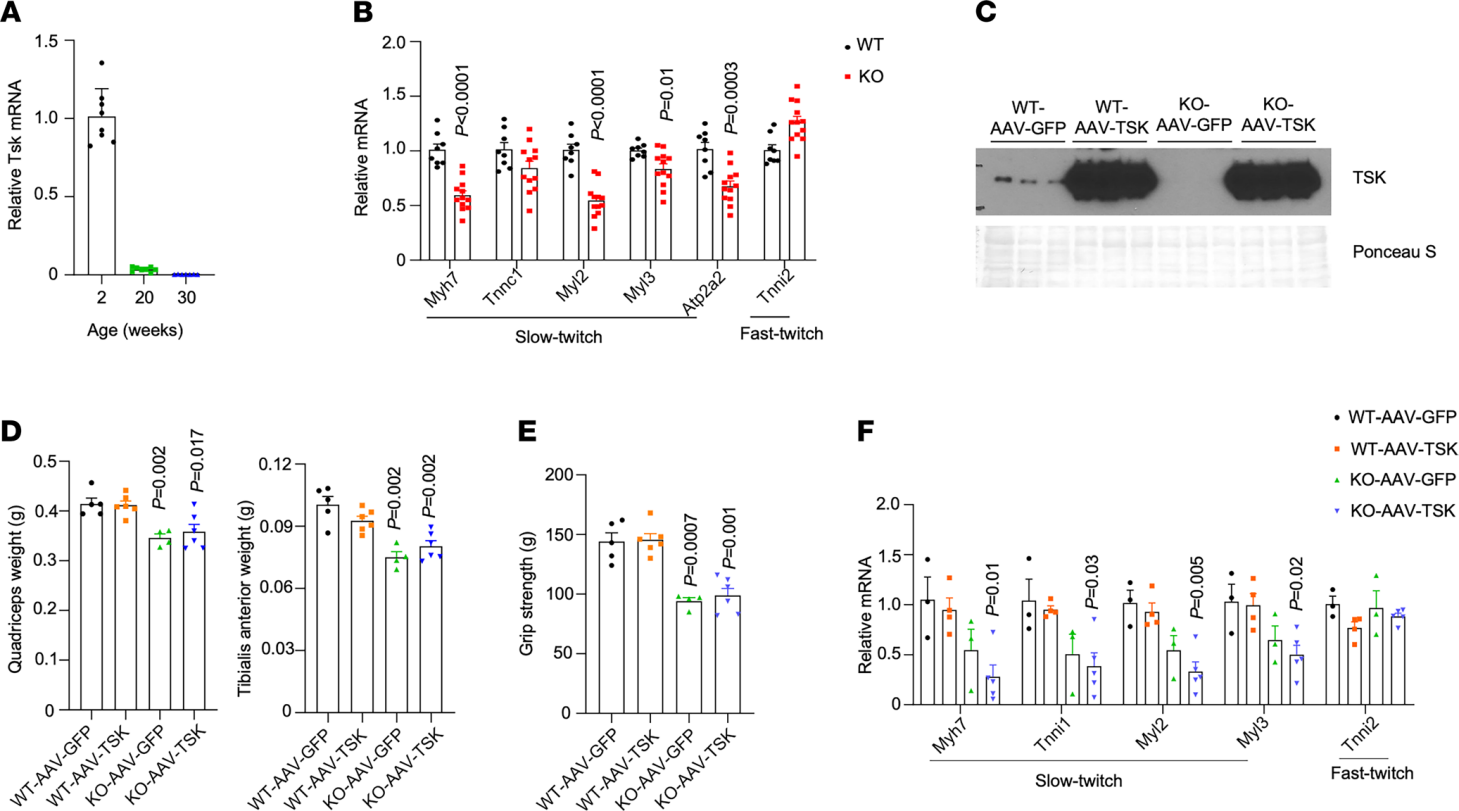
Effects of AAV-mediated TSK overexpression on skeletal muscle. (**A**) qPCR analysis of Tsk expression in quadriceps in mice at 2, 20, or 30 weeks of ages. (**B**) qPCR analysis of quadriceps gene expression in WT (*n* = 8) and TSK-KO (*n* = 12) mice at 2 weeks of age. (**C**) Immunoblots of plasma samples from WT and TSK-KO mice transduced with AAV-GFP or AAV-TSK. Ponceau S staining is included as loading control. (**D**) Quadriceps and TA muscle weight in mice 3 weeks following AAV transduction. (**E**) Forelimb grip strength. (**F**) qPCR analysis of muscle gene expression in transduced mice. Data represent mean ± SEM and were analyzed by 2-tailed unpaired Student’s *t* test.

TSK is abundantly expressed in the liver, which provides the main source of TSK in circulation. TSK expression has also been detected in other tissues and the CNS, suggesting that local production of TSK may play an important role in mediating its biological effects. We examined skeletal muscle TSK gene expression at different stages of postnatal growth. While TSK mRNA expression was readily detectable in quadriceps from mice at 2 weeks of age, its expression markedly decreased and remained barely detectable in adult mice (Figure 5A). In contrast, hepatic TSK expression and plasma levels showed a strong increase in adult mice compared with young pups (Supplemental Figure 2). These findings suggest that a switch from local production to endocrine TSK occurs in skeletal muscle during postnatal growth. Interestingly, reduced slow-twitch gene expression was already apparent at 2 weeks of age, suggesting that TSK deficiency impaired myofiber gene expression during embryonic and/or early postnatal periods (Figure 5B). To assess whether reexpression of TSK is sufficient to restore myofiber gene expression in adult mice, we generated a recombinant AAV vector to elevate circulating TSK to supraphysiological levels and assess its effects on muscle phenotype (Figure 5C). Consistently, we observed reduced muscle mass, forelimb grip strength, and myofiber gene expression in TSK-KO mice (Figure 5, D–F). Compared with control AAV-GFP, AAV-TSK failed to rescue these defects in TSK-KO mice. These results suggest that TSK may exert its effects on myofiber gene expression and function at early stages of muscle development.

### Muscle regeneration is impaired in TSK-deficient mice.

**Figure 6:**
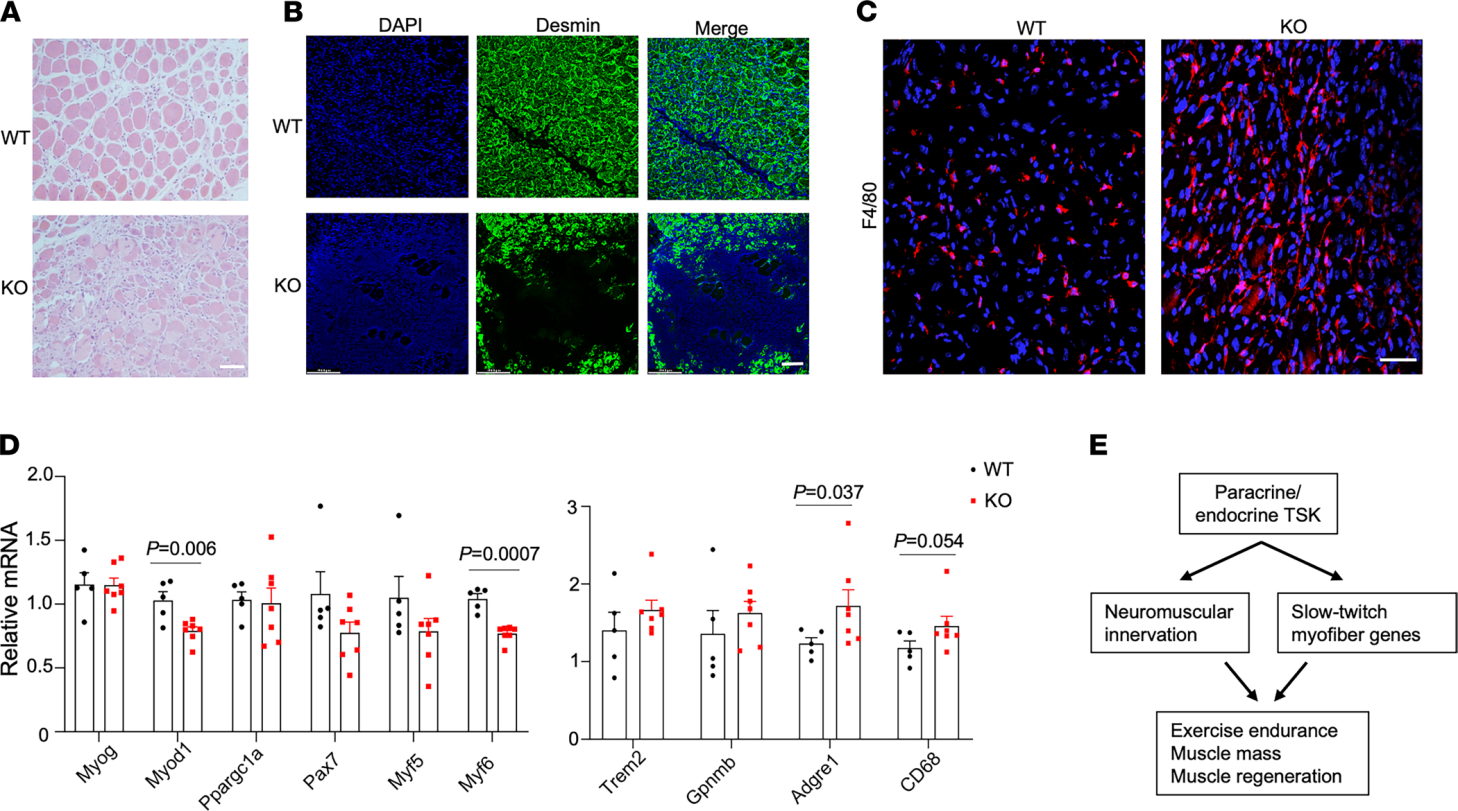
TSK deficiency impairs muscle regeneration following cardiotoxin-induced injury. (**A**) H&E staining of TA muscle from WT and TSK-KO mice 8 days following intramuscular cardiotoxin treatment. (**B**) Anti-desmin immunofluorescence staining of TA muscle sections from treated mice. (**C**) F4/80 immunofluorescence staining. (**D**) qPCR analysis of muscle gene expression. (**E**) A schematic diagram illustrating the role of TSK in muscle development and function. Data in **D** represent mean ± SEM and were analyzed by 2-tailed unpaired Student’s *t* test. Scale bars: 100 µm.

Having established that TSK is required for maintaining myofiber gene expression and function, we next examined whether it plays a role in muscle regeneration. TSK has been shown to physically interacts with extracellular signaling molecules such as TGF-β and FGF (24). As such, its deficiency may impair the program of muscle regeneration following acute muscle injury. To test this, we performed intramuscular injection of cardiotoxin to induce muscle injury and examined muscle histology and gene expression 8 days following injection. As expected, cardiotoxin treatment resulted in severe damage to myocytes and elicited a strong local inflammatory response as indicated by leukocyte infiltration (Figure 6A). Compared with control, myofiber damage and infiltration of immune cells remained evident 8 days following the onset of cardiotoxin-induced injury, as revealed by histological and Desmin immunofluorescence staining (Figure 6, A and B). Consistently, we observed increased infiltration of F4/80^+^ macrophages in injected muscle from TSK-deficient mice (Figure 6C). This defective muscle regeneration was associated with reduced expression of myogenic regulators (*Myod1*, *Myf6*) and elevated expression of macrophage markers (*F4/80*, *CD68*) (Figure 6D). These results support a critical role of TSK in facilitating muscle regeneration and tissue homeostasis. To our surprise, AAV-mediated overexpression of TSK failed to improve muscle injury and regeneration in this model (Supplemental Figure 3), suggesting that developmental TSK signaling may account for its protective effects.

## Discussion

Muscle mass and its contractile and metabolic properties represent 2 major aspects of myofiber biology underlying the endurance capacity of skeletal muscle. Several growth factors have been implicated in the regulation of myofiber growth and muscle mass. Insulin-like growth factor-I (IGF-I) is a prototypical growth factor that promotes muscle growth and hypertrophy (25), while Myostatin is a member of the TGF-β superfamily of ligands (26) and exerts an inhibitory effect on muscle mass regulation. Recent studies have also implicated a member of the FGF family of signaling ligands, FGF19, in the regulation of skeletal mass (27). However, our understanding of the extracellular signals that shape the contractile and metabolic phenotype of myofibers remain limited. In this study, we explored the role of TSK in myofiber specification and muscle function. TSK-deficient mice exhibited reduced muscle mass that is linked to notable defects in the expression of contractile genes characteristic of slow-twitch muscle fibers, leading to reduced muscle strength and endurance capacity. The highly specific effects of TSK deficiency on the contractile, but not metabolic, aspect of the myofiber phenotype raise the possibility that TSK may act on a yet-to-be defined signaling pathway in promoting muscle-nerve coupling and slow-twitch muscle fiber formation (Figure 6E).

Perhaps the most intriguing aspect of TSK in myofiber regulation is its indispensable and highly specific role in maintaining the expression of slow-twitch contractile genes. In fact, our RNA-Seq analysis failed to detect significant changes in metabolic gene expression, particularly those involved in mitochondrial biogenesis and oxidative metabolism, in skeletal muscle from TSK-KO mice. This is somewhat surprising, given that the metabolic properties of myofibers are tightly linked to their contractile functions, as observed in mice with transgenic PGC-1α overexpression in skeletal muscle (28). However, uncoupling of contractile and metabolic gene programs has been previously reported. For example, BAF60c is a regulator of muscle glycolytic metabolism that elicits modest effects on muscle fiber type regulation (29). As such, it is possible that TSK deficiency predominantly perturbs regulatory pathways that govern slow-twitch myofiber gene expression. The impairments of myofibrillar and contractile gene expression likely underlie the diminished exercise capacity in TSK-KO mice; however, it cannot be fully ruled out that altered systemic and muscle energy metabolism and cardiac function may contribute to the phenotype. Whether defective TSK signaling contributes to loss of muscle mass and exercise capacity under disease conditions in humans remains an important area for future exploration.

The exact molecular mechanisms through which TSK regulates muscle fiber gene expression and function remain currently unknown. It is possible that diminished alignment of nerve fibers with neuromuscular junctions may impair transduction of motor nerve firing, which is essential for shaping diverse aspects of myofiber biology. Previous studies have demonstrated that TSK physically interacts with extracellular signaling molecules such as the TGF-β, FGF, and Wnt families of ligands (14–16). As such, it is possible that TSK may regulate muscle gene expression indirectly through modulating bioavailability and/or activity of these developmental regulators. While high levels of TSK expression have been detected in the liver, its expression has been observed in multiple tissues, including the CNS, adipose tissue, and skeletal muscle at certain developmental stages. The relative contributions from endocrine versus paracrine TSK from local sources in the regulation of muscle gene expression remain unknown. However, given that AAV-mediated TSK overexpression failed to rescue the myofiber phenotype in adult TSK-KO mice, it is likely that local TSK secretion may play a more dominant role during certain developmental stages. Together, our work opens up these important questions that may shed new light on the signaling mechanisms that govern TSK action in skeletal muscle.

## Methods

### Animal studies.

Mice were maintained under 12-hour L/D cycles with free access to food and water. For chow feeding, mice were fed Teklad 5001 laboratory rodent chow diet. For HFD feeding, mice were fed a diet containing 60% of calories from fat (D12492, Research Diets Inc.) starting at 3 months of age. The generation of TSK-KO mice was previously described (30). To induce muscle injury, mice received a single i.m. injection (TA) of 50 μL of 10 μM cardiotoxin. Muscle injury and regeneration were analyzed 1 week after the treatment. For recombinant AAV virus transduction, AAV8-CAG-GFP or AAV8-CAG-TSK (1 × 10^11^ genomic copies per mouse) was delivered via tail vein injection. Forelimb grip strength in WT and TSK-KO mice was measured 3 weeks after AAV transduction. All experiments were performed on male mice unless otherwise indicated.

### Histological analysis.

Skeletal muscle samples were embedded with optimal cutting temperature (OCT) compound and immediately frozen with liquid nitrogen–cooled isopentane following dissection. SDH and α-GPDH staining were performed as previously described (31). For immunofluorescence staining, cryosections of muscle were fixed with 4% paraformaldehyde (PFA), blocked with 10% goat serum, and incubated with antibodies against MHC-I (1:200, BA-F8, Developmental Studies Hybridoma Bank [DSHB]), MHC-IIa (1:200, SC-71, DSHB), MHC-IIb (1:200, BF-F3, DSHB), Desmin (1:400, RB-9014-P, Thermo Fisher Scientific), and F4/80 (1:200, MCA497G, Bio-Rad) at 4°C overnight, followed by incubation with Alexa Fluor–conjugated secondary antibody (1:300, Invitrogen) for 1 hour. After washing and incubation with DAPI for 1 minute, stained sections were mounted with mounting medium and imaged on a Nikon A1 confocal microscope. Myofiber sizes were calculated based on WGA lectin immunofluorescence staining of TA muscles. ImageJ (NIH) was used to measure the cross-section area of individual WGA lectin-stained myofibers.

For neuromuscular junction staining, TA muscle was fixed with 4% PFA for 20 minutes, washed with PBS, and then torn into small pieces in the direction of the tendon. Small pieces were washed with PBS plus 1% Triton-X100 for 2 hours and treated with blocking buffer (5% normal goat serum in PBS with 0.5% Triton-X100) for 4 hours. Samples were incubated with antibodies against neruofilament (1:500, 2837S, Cell Signaling Technology) and synapsin (1:500, 5297S, Cell Signaling Technology) at 4°C overnight. The stained muscle fibers were washed with PBS and subsequently incubated with Alexa Fluor–conjugated secondary antibody and CF594-labeled α-BTX (1:1000, 00007, Biotium) for 1 hour. Samples were mounted with mounting medium and imaged using a confocal microscope. Innervation of acetylcholine receptors (AChRs) was defined as colocalization of axon terminals with CF594-conjugated α-BTX labeled AChRs.

### Gene expression analysis.

Total RNA from muscle tissues was extracted using TRIzol method. For qPCR analysis, 2 μg of total RNA was reverse transcribed using MMLV-RT followed by qPCR using SYBR Green (Thermo Fisher Scientific). Quadriceps muscle RNA-Seq was performed using Illumina HiSeq 4000 at BGI Genomics. RNA-Seq data were analyzed using DESeq2 and deposited into the NCBI Gene Expression Omnibus (GEO) database (accession no. GSE193089).

### Western blot analysis.

Total lysates were prepared in a lysis buffer containing 50 mM Tris (pH 7.5), 150 mM NaCl, 5 mM NaF, 25 mM β-glycerol phosphate, 1 mM dithiothreitol (DTT), and freshly added protease inhibitors. The lysates were separated by SDS-PAGE and transferred to the PVDF membrane, followed by incubation with first antibodies and secondary antibodies. Rabbit polyclonal TSK antibodies were generated using mouse TSK peptides and were affinity purified before use. Additional antibodies used in this study were: Myh4 (BF-F3, DSHB), Myh7 (BA-F8, DSHB), Myoglobin (sc-74525, Santa Cruz Biotechnology Inc.), and Hsp90 (sc-7947, Santa Cruz Biotechnology Inc.). Full, uncut images of the immunoblots are provided in the online Supplemental Material

### Treadmill running.

We performed a high-intensity treadmill test using a motorized, speed-controlled treadmill system (Columbus Instruments). The angle was set to a 10% incline. Before performing the treadmill running experiment, mice were trained for running on a treadmill for 10 minutes at 8 m/min 3 times in 1 week. For treadmill running, the running speed was raised to 6 m/min, and it increased by 2 m/min every 5 minutes to a maximal pace of 30 m/min. WT and TSK-KO mice were allowed to run until exhaustion for the measurements of total running time and distance. Exhaustion was determined when the mice spent more than 5 seconds on an electric shocker without resuming running. The pace at which exhaustion occurs was recorded as the maximal speed. Blood lactate levels were measured immediately after exhaustion using a Lactate Pro blood lactate test meter (ARKRAY Inc.), WT and TSK-KO mice were subjected to running for 10 minutes. Mice were anesthetized, and skeletal muscle were harvested for the measurements of muscle glycogen content.

### Grip strength.

Forelimb grip strength of mice was measured using Grip Strength Meter (Columbus Instruments). Mice were held by the tail and allowed to grab the grid with only their forepaws before being pulled in a parallel direction. The average of the highest 3 measurements from 5 strength measurements was recorded as grip strength.

### Muscle glycogen measurement.

Muscle tissues (50 mg of tissue) were dissolved in 200 μL of 30% (wt/vol) KOH at 98°C for 30 minutes. 67 μL of 1M Na_2_SO_4_ and 535 μL of ethanol were added to the muscle homogenate, followed by heating at 98°C for 5 minutes and centrifugation at 16,000*g* for 5 minutes at room temperature. The pellet was resuspended in 100 μL of H_2_O. In total, 200 μL of ethanol was then added before centrifugation at 16,000*g* for 5 minutes at room temperature. Until being completely dried, the pellet was resuspended in 100 μL of 1 mg/mL Amyloglucosidase (MilliporeSigma) and incubated at 37°C for 3 hours. Supernatants were collected for glucose assay using the Autokit Glucose (FUJIFILM).

### Statistics.

Statistical analysis was performed using GraphPad Prism 8. Two-tailed Student’s *t* test was used to analyze the differences between 2 groups, 1-way ANOVA followed by post hoc Tukey’s test was used to determine the differences among multiple groups, and 2-way ANOVA followed by Tukey’s multiple-comparison test was used for 2 variables. *P* values of less than 0.05 were considered statistically significant.

### Study approval.

All animal studies were performed according to the procedures approved by the University of Michigan IACUC.

## Author contributions

JDL and QW conceived the project and designed research. QW, XQ, TL, and CA performed the studies and analyzed the data. QW, JDL, and JFH prepared the manuscript.

## Supplementary Material

Supplemental data

## Version 1 01/13/2022

In-Press Preview

## Version 2 02/22/2022

Electronic publication
