# Supplementary material for: The hepatokine TSK maintains myofiber integrity and exercise endurance and contributes to muscle regeneration
Source: JCI Insight. 2022 Feb 22;7(4):e154746. doi: 10.1172/jci.insight.154746 (PMC8876464; doi:10.1172/jci.insight.154746)
Supplement: Supplemental data [file jciinsight-7-154746-s060.pdf]

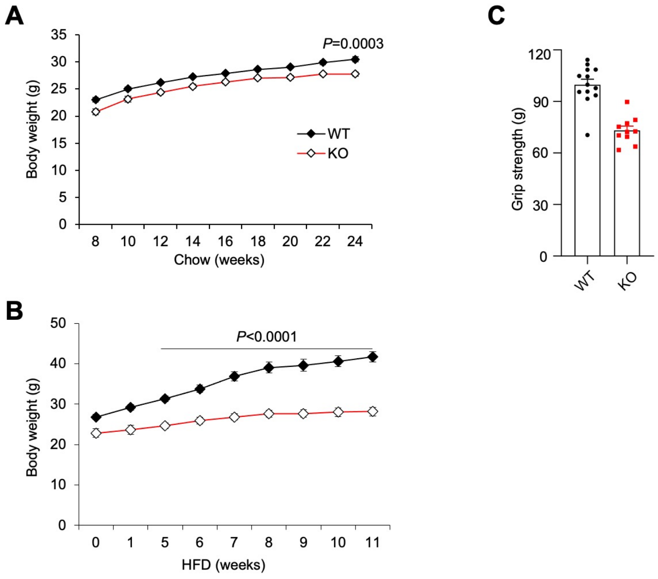

Figure S1. Growth curve and grip strength.

(A) Growth curve of WT (n=8) and TSK KO (n=10) mice fed standard rodent chow. **(B)** Growth curve of WT (n=8) and TSK KO (n=7) mice fed HFD. **(C)** Grip strength in a second cohort of WT (n=13) and TSK KO (n=10) mice. Data represent mean ± SEM and were analyzed by two-tailed unpaired Student's *t*-test.

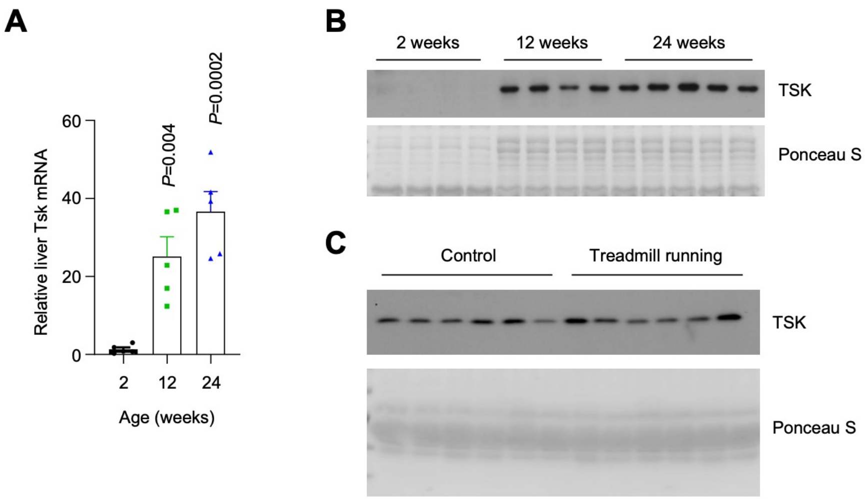

Figure S2. Postnatal regulation of hepatic TSK expression and its plasma levels.

(A) qPCR analysis of TSK mRNA expression in mouse liver at different ages. **(B)** Immunoblot of plasma TSK. **(C)** Immunoblot of plasma TSK in HFD-fed mice without or with daily 30-minute treadmill running for a total of 20 days. Data in (**A**) represent mean ± SEM; two-tailed unpaired Student's *t*-test.

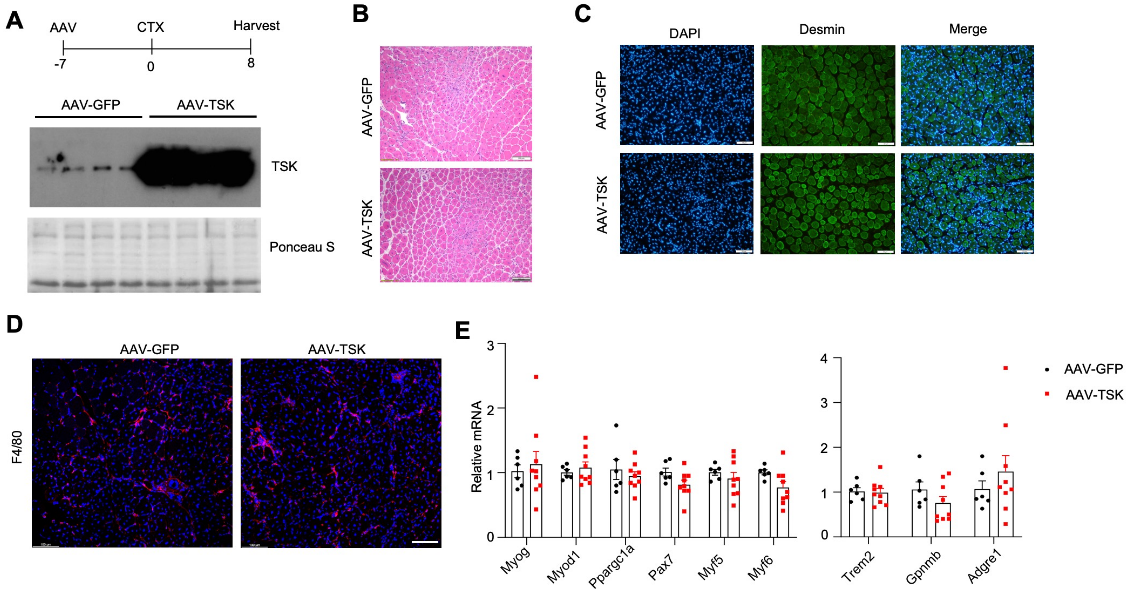

Figure S3. Effects of AAV-mediated TSK overexpression on muscle injury and repair. **(A)** AAV-mediated TSK overexpression in mice followed by intramuscular cardiotoxin treatment. **(B)** H&E staining of TA muscle in mice transduced with AAV-GFP (n=6) or AAV-TSK (n=9). **(C)** Anti-Desmin immunofluorescence staining of TA muscle sections from treated mice. **(D)** F4/80 immunofluorescence staining. **(E)** qPCR analysis of muscle gene expression. Data in (**E**) represent mean ± SEM and were analyzed by two-tailed unpaired Student's *t*-test.
